# A machine-learning-derived online prediction model based on inflammatory and nutritional composite indicators for acute kidney injury in sepsis patients with multiple myeloma

**DOI:** 10.3389/fnut.2026.1874801

**Published:** 2026-09-07

**Authors:** Qin Zhang, Qi Xin, Huayan Guo

**Affiliations:** 1 Department of Hematology, Xi’an No. 3 Hospital, The Affiliated Hospital of Northwest University, Xi’an, Shaanxi, China; 2 Department of Emergency Surgery, Shaanxi Provincial People’s Hospital, Xi’an, China

**Keywords:** acute kidney injury, machine learning, multiple myeloma, sepsis, SHAP

## Abstract

**Background:**

Acute kidney injury (AKI) frequently complicates sepsis in patients with multiple myeloma (MM), yet no validated predictive tool integrates the inflammatory and nutritional disturbances specific to this dual-disease population. We aimed to develop and externally validate an interpretable machine learning (ML) model—based on composite inflammatory-nutritional indices—to predict in-hospital AKI in septic patients with MM, and to deploy it as a web-based point-of-care tool.

**Methods:**

In this retrospective cohort study, 362 septic MM patients (development cohort: 253 training, 109 internal test) and 71 temporal validation patients were enrolled from Shaanxi Provincial People’s Hospital (2020–2025). Moreover, an independent external validation cohort from a separate tertiary hospital (*n* = 118, Xi’an No. 3 Hospital). Candidate predictors included 8 composite indices (e.g., NLR, SII, LAR, AAPR) alongside routine clinical and laboratory variables. Boruta and recursive feature elimination (RFE) identified a parsimonious predictor set. Six ML algorithms (logistic regression, SVM, MLP, LightGBM, XGBoost, Random Forest) were compared using AUC, calibration, and decision curve analysis. The final model was interpreted with SHAP and implemented as a public Streamlit application.

**Results:**

AKI occurred in 206/362 (56.9%) patients. Eight predictors were selected: NLR, LAR, creatinine, SII, PTA, cystatin C, CRP, and AAPR. Random Forest demonstrated the most robust and generalizable performance, achieving a temporal validation AUC of 0.8574 (accuracy 0.7465, sensitivity 0.8205, specificity 0.6562). Moreover, Random Forest also achieved an external validation AUC of 0.9650 (accuracy 0.8983, sensitivity 0.9000, specificity 0.8971), with favorable calibration and consistent net clinical benefit. SHAP analysis identified higher NLR, LAR, SII, cystatin C, creatinine, and CRP, together with lower PTA and AAPR, as the most influential predictors in the model. The model is freely available at (https://gftcs94ast7omb44cg2izq.streamlit.app/).

**Conclusion:**

An interpretable Random Forest model employing eight routinely available indicators accurately predicts AKI in septic MM patients, bridging algorithmic complexity and bedside applicability. This tool may facilitate early risk stratification and personalized renal protection in this high-risk group.

## Introduction

Sepsis remains a leading cause of morbidity and mortality in critically ill patients worldwide, and acute kidney injury (AKI) represents one of its most severe and frequent complications, occurring in approximately 40–50% of septic patients and independently conferring a substantially increased risk of adverse outcomes (1). The pathogenesis of sepsis-associated AKI is multifactorial, involving systemic inflammation, microvascular dysfunction, metabolic reprogramming, and immune dysregulation. Among the vulnerable populations, patients with multiple myeloma (MM) constitute a particularly high-risk subgroup. MM is characterized by the clonal proliferation of plasma cells and the overproduction of monoclonal immunoglobulins, which directly predispose the renal tubules to cast nephropathy, tubular injury, and interstitial inflammation (2, 3). When sepsis supervenes, the synergistic nephrotoxic insults—hemodynamic instability, exposure to nephrotoxic agents, and the pre-existing malignant milieu—dramatically amplify the likelihood of AKI. Despite its clinical importance, robust and individualized risk prediction tools specifically tailored to septic patients with MM are currently lacking.

Traditional statistical models and generic critical care scores, such as the Sequential Organ Failure Assessment (SOFA) score, offer only a limited capacity to capture the complex, nonlinear interactions between inflammatory markers, nutritional status, and renal vulnerability in this dual-disease population. Widely used clinical risk scores were originally derived from general ICU cohorts and may fail to account for the distinct immunological and metabolic derangements that characterize MM. Consequently, there is an unmet need for a more precise, data-driven approach capable of integrating multidimensional information to identify patients at the highest risk of AKI at an early, potentially modifiable stage.

Machine learning (ML) has emerged as a powerful tool for clinical prediction by leveraging high-dimensional data to uncover intricate patterns beyond the reach of conventional regression (4, 5). Importantly, composite indicators that synthesize information from routine laboratory parameters—such as the neutrophil-to-lymphocyte ratio (NLR), the systemic immune-inflammation index (SII), and albumin-based nutritional indices—have shown promise as surrogates for the host inflammatory and nutritional state, and have been linked to adverse outcomes in sepsis and hematological malignancies (6–8). However, to our knowledge, no study has systematically evaluated the predictive value of these composite inflammatory-nutritional indices for AKI in septic patients with MM, and no interpretable ML-based tool has been translated into a clinically deployable format for this specific indication.

To fill this gap, we aimed to (1) develop and compare multiple ML algorithms to predict in-hospital AKI in septic patients with MM, (2) identify the most informative inflammatory and nutritional composite predictors using rigorous feature selection, and (3) construct an interpretable, web-based prediction model deployed via Streamlit to provide individualized risk estimates at the bedside. By integrating explainability through Shapley Additive Explanations (SHAP), our framework seeks to bridge the gap between algorithmic complexity and clinical transparency, facilitating early risk stratification and targeted renal protective strategies in this high-risk patient population (9, 10).

## Materials and methods

### Study design and population

This retrospective study was carried out at Shaanxi Provincial People’s Hospital and received approval from the Institutional Review Board (approval number: 2025R082). To assess generalizability, an independent external validation cohort was retrospectively collected from Xi’an No. 3 Hospital. The use of data from Xi’an No. 3 Hospital was approved by its institutional review board (approval number: SYLL-2026-104). Given the study’s retrospective design, the requirement for written informed consent was waived.

We screened all adult patients (≥18 years of age) who were hospitalized with a diagnosis of sepsis and pre-existing MM. Sepsis was identified using the Sepsis-3 criteria—namely, a suspected or confirmed infection accompanied by an acute rise of ≥2 points in the SOFA score (11). The diagnosis of MM was established in accordance with the International Myeloma Working Group (IMWG) updated diagnostic criteria, which require the presence of ≥10% clonal bone marrow plasma cells or a biopsy-proven plasmacytoma, accompanied by one or more myeloma-defining events—either attributable CRAB features (hypercalcemia, renal failure, anemia, bone lesions) or SLiM biomarkers (≥60% clonal bone marrow plasma cells, involved/uninvolved serum free light chain ratio ≥100, or >1 focal lesion on MRI) (12, 13).

Individuals admitted between January 2020 and October 2024 were assigned to the internal development cohort, while those hospitalized from November 2024 to December 2025 served as a temporal validation cohort from Shaanxi Provincial People’s Hospital. Furthermore, External validation set from Xian No. 3 Hospital between November 2023 and December. Exclusion criteria were: (1) age ≤18 years old; (2) the length of stay in hospital <24 h; (3) end-stage renal disease or current hemodialysis, (4) AKI present at admission or occurring within the first 24 h after admission; and (5) incomplete key laboratory or outcome data.

### Data collection

Demographic data, vital signs at admission, comorbidities, and in-hospital interventions were extracted from the electronic medical record system. Laboratory parameters measured within the first 24 h of admission were collected, including coagulation profiles (prothrombin time activity [PTA], prothrombin time [PT], activated partial thromboplastin time [APTT], thrombin time [TT], international normalized ratio [INR], fibrin degradation products [FDP], D-dimer, fibrinogen [FIB]), inflammatory markers (C-reactive protein [CRP], procalcitonin [PCT]), blood cell counts (white blood cells [WBC], neutrophils, lymphocytes, monocytes, platelets, red blood cells [RBC], hemoglobin), and biochemical indices (albumin, globulin, bilirubin, alanine aminotransferase, aspartate aminotransferase, glucose, uric acid, cystatin C, blood urea nitrogen [BUN], creatinine [Cr], total cholesterol). Disease severity was assessed by the SOFA score. In addition to the composite indices, baseline estimated glomerular filtration rate (eGFR) was calculated using the CKD-EPI (Chronic Kidney Disease Epidemiology Collaboration) equation based on the baseline serum creatinine measured within the first 24 h. Pre-existing chronic kidney disease (CKD) was defined as an eGFR <60 mL/min/1.73 m^2^ or a documented history of CKD in the medical records. These variables were incorporated into the descriptive baseline comparison but were not included as candidate predictors in the model to avoid redundancy with creatinine and cystatin C.

In addition, a panel of composite indicators reflecting the inflammatory, nutritional, and immune status was calculated from the laboratory data obtained within the first 24 h of admission. The formulas were defined as follows: Neutrophil-to-lymphocyte ratio (NLR) = neutrophil count (×10^9^/L)/lymphocyte count (×10^9^/L); Platelet-to-lymphocyte ratio (PLR) = platelet count (×10^9^/L)/lymphocyte count (×10^9^/L); Lymphocyte-to-monocyte ratio (LMR) = lymphocyte count (×10^9^/L)/monocyte count (×10^9^/L); Systemic immune-inflammation index (SII) = platelet count (×10^9^/L) × neutrophil count (×10^9^/L)/lymphocyte count (×10^9^/L); Systemic inflammation response index (SIRI) = neutrophil count (×10^9^/L) × monocyte count (×10^9^/L)/lymphocyte count (×10^9^/L); Prognostic nutritional index (PNI) = serum albumin (g/L) + 5 × total lymphocyte count (×10^9^/L); Albumin-alkaline phosphatase ratio (AAPR) = serum albumin (g/L)/alkaline phosphatase (U/L); Lactate-albumin ratio (LAR) = serum lactate (mmol/L)/serum albumin (g/L). Continuous renal replacement therapy (CRRT) and mechanical ventilation during hospitalization were also recorded.

We acknowledge that detailed MM-specific parameters, such as serum free light chain (sFLC) levels, immunofixation electrophoresis, bone marrow plasma cell infiltration, and specific antimyeloma regimens, are central to the pathogenesis of myeloma-related kidney injury. However, these parameters are often not routinely available within the first 24 h of emergency admission or ICU triage in real-world practice, which would preclude their use for early, point-of-care risk stratification. Therefore, the present study deliberately focused on universally accessible laboratory indices obtained within the initial 24-h window to maximize clinical deployability. We also did not incorporate infectious source or vasopressor use as candidate predictors because these are dynamic management variables rather than baseline risk attributes, and their effects on renal injury are primarily mediated through the systemic inflammatory and hemodynamic pathways already captured by our composite indices (e.g., LAR, CRP, SOFA components).

### Outcome definition

The primary outcome was the occurrence of AKI during the index hospitalization. AKI was defined according to the kidney disease: Improving Global Outcomes (KDIGO) criteria, i.e., an increase in serum Cr by ≥0.3 mg/dL (≥26.5 μmol/L) within 48 h, or a rise to ≥1.5 times the baseline value known or presumed to have occurred within the prior 7 days (14). The reference serum creatinine for KDIGO criteria was defined as the first measurement obtained within 24 h of hospital admission, which served as the starting point for detecting subsequent acute changes. We acknowledge that this value may not represent the patient’s true pre-admission baseline renal function, particularly in MM patients with pre-existing chronic kidney disease or monoclonal gammopathy-related renal impairment. To ensure a clear temporal relationship between predictors and outcome and to predict *de novo* AKI, patients who already met KDIGO criteria for AKI at admission or within the first 24 h were excluded from the analysis. Due to the inherent limitations of retrospective data collection, where urine output records were often incomplete and confounded by diuretic use, the urine-output criterion of the KDIGO definition was not employed; this approach is consistent with previous large-scale retrospective studies.

### Feature selection

To select the most relevant predictors while keeping the model parsimonious and avoiding data leakage, we performed a two-step variable selection procedure exclusively on the training set. First, the Boruta algorithm—a random forest-based method—was applied to all candidate variables; it creates shadow copies of each variable and retains only those original features whose importance is statistically greater than that of their shadow counterparts. Second, recursive feature elimination (RFE) was used to determine the optimal subset size by iteratively removing the least important variables based on the AUC. The final predictor set was chosen as the combination that achieved the highest AUC with the fewest features. Of note, because CRRT is a therapeutic intervention initiated after AKI onset rather than a baseline predictor, its inclusion would introduce reverse causality and information leakage; therefore, CRRT was removed from the candidate pool prior to RFE. The combination of variables that yielded the highest AUC with the fewest predictors was selected for downstream model development.

It should be noted that univariate significance does not guarantee retention in the Boruta-RFE pipeline, as these algorithms evaluate each variable’s conditional contribution in a multivariate context. Variables that convey information largely overlapping with other selected predictors—or that are distal antecedents rather than direct pathophysiological mediators—may be excluded despite showing marginal associations.

### Development and validation of machine learning models

Six supervised machine learning algorithms were developed to predict in-hospital AKI: Logistic Regression (LR), Support Vector Machine (SVM), Multilayer Perceptron (MLP), Light Gradient Boosting Machine (LightGBM), Extreme Gradient Boosting (XGBoost), and Random Forest (RF). Hyperparameters for each algorithm were optimized via a grid search combined with five-fold cross-validation on the training set, with the area under the receiver operating characteristic curve (AUC) serving as the selection criterion. All models were implemented in Python (version 3.12.5) using the scikit-learn, XGBoost, and LightGBM libraries. The final models were evaluated on the internal test set and the temporal validation cohort, and external validation cohort.

Hyperparameter tuning was performed using a five−fold cross−validation combined with grid search on the training set. The final hyperparameters for each model were selected based on the highest cross−validated AUC. All models were implemented using Python 3.12.5 with the scikit−learn, XGBoost, and LightGBM libraries. The best parameters for each model are as follows: LR (C: 10, penalty: l2, solver: saga); RF (max_depth: 10, min_samples_split: 10, n_estimators: 200); SVM (C: 1, gamma: scale, kernel: linear); XGB (learning_rate: 0.1, max_depth: 3, n_estimators: 100, subsample: 0.8); LightGBM (boosting_type: gbdt, learning_rate: 0.01, n_estimators: 200, num_leaves: 31); MLP (hidden_layer_sizes: 50, learning_rate_init: 0.001, max_iter: 200). The optimal parameter configurations obtained through Grid Search effectively mitigated the risk of overfitting and enhanced the predictive performance of each machine learning model on the dataset.

### Model evaluation

Model performance was assessed from three dimensions: discrimination, calibration, and clinical utility. Discrimination was quantified by the AUC, supplemented by accuracy, sensitivity, specificity, precision, and F1 score. Calibration was examined through calibration plots and the Brier score, with a lower Brier score indicating better correspondence between predicted probabilities and observed outcomes. Clinical utility was appraised by decision curve analysis (DCA), which estimates the net benefit of the model across a continuum of threshold probabilities. All metrics were calculated on the training set (cross-validated), internal test set, and external validation cohort to ensure robustness and generalizability.

To benchmark the incremental clinical value of our model, we compared its discriminative performance against the SOFA score alone. The SOFA score was treated as a single continuous predictor, and its AUC was calculated in the temporal and external validation cohorts. The difference in AUCs between the Random Forest model and the SOFA score was tested using DeLong’s method for paired AUC comparison, with a two-sided *p* value <0.05 considered statistically significant.

### Model interpretability

To enhance transparency and foster clinical trust, Shapley Additive Explanations (SHAP) were applied to the top-performing model. SHAP values decompose each prediction into the contribution of individual features, enabling both global interpretation (via summary plots that depict the direction and magnitude of feature effects) and local interpretation (via force plots that explain predictions for individual patients). Feature importance rankings derived from SHAP were compared with those from logistic regression to confirm consistency.

### Deployment of an online prediction tool

To enable real-time, point-of-care risk assessment, the final Random Forest model was deployed as an interactive, publicly accessible web application. The front-end interface was constructed using Streamlit, an open-source Python library designed for rapid development of clinical decision-support tools. The serialized model pipeline was uploaded and served via Streamlit Community Cloud, which automatically redeploys upon code updates. The complete source code, including data preprocessing routines and the trained model, was version-controlled and is openly available on GitHub, ensuring full reproducibility and transparency. Through this interface, clinicians can enter the values of the eight selected predictors and immediately obtain an individualized probability of AKI, accompanied by a SHAP-based force plot that visualizes the direction and magnitude of each feature’s contribution to the risk estimate.

### Statistical analysis

Variables that followed a normal distribution were presented as mean ± standard deviation (SD) and compared between groups using the independent samples *t*-test. Variables that deviated from a normal distribution were summarized as medians with interquartile ranges (IQR) and analyzed with the Mann–Whitney *U* test. Categorical variables were expressed as frequencies with percentages and compared between groups using the chi-squared test or Fisher’s exact test, as appropriate.

All statistical tests were two-sided, and a *p* value of less than 0.05 was considered statistically significant. Data analyses were performed using SPSS version 27.0 (IBM Corp.) and Python version 3.12.5.

## Results

### Baseline characteristics of the study population

**Figure 1:**
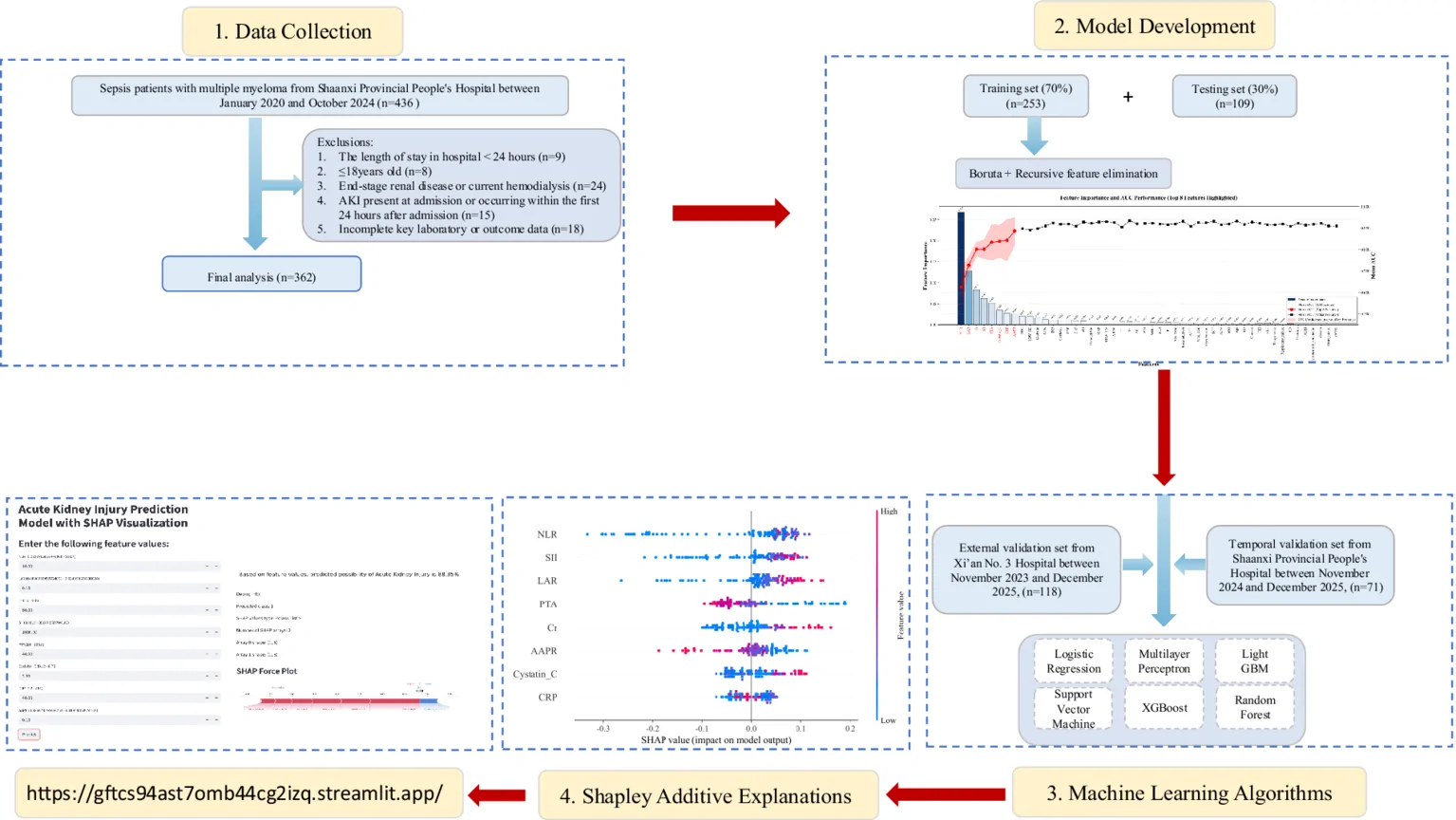
Flowchart illustrating the inclusion of study subjects and the dataset splitting process.

The participant selection flow is depicted in Figure 1. Of the initial screened patients, 74 were excluded: 8 were aged ≤18 years; 9 had a length of stay <24 h; 24 had end-stage renal disease or were on chronic hemodialysis; 15 met KDIGO criteria for AKI at admission or within the first 24 h; 10 had incomplete key laboratory data (with missing variables distributed as cystatin C [*n* = 5], CRP [*n* = 3], PTA [*n* = 1], and creatinine [*n* = 1]); and 8 had incomplete outcome data, of whom 5 lacked follow-up serum creatinine measurements during hospitalization and 3 were transferred to or discharged from other facilities before AKI could be evaluated. No patient had more than one missing predictor or outcome component. After applying complete-case analysis, the final cohort comprised 362 patients with complete data for all candidate variables and outcomes. As shown in Supplementary Table 1S, the AKI incidence was 56.9% in both the training (144/253) and internal test (62/109) cohorts, 54.9% (39/71) in the temporal validation cohort, and 42.4% (50/118) in the external validation cohort. Across all cohorts, KDIGO stage 1 accounted for the majority of AKI events (52.8–54.0%), followed by stage 2 (25.6–26.4%) and stage 3 (20.0–21.0%). The median time from admission to AKI onset was 5 days (IQR: 3–8 days) in the development and temporal validation cohorts, and 6 days (IQR: 3–9 days) in the external validation cohort. All patients who met KDIGO criteria within the first 24 h were excluded, ensuring that the reported timing reflects *de novo* AKI occurring after the baseline predictor assessment window.

Table 1 summarizes the demographic, clinical, and laboratory characteristics stratified by AKI status. Patients in the AKI group were slightly older, though the difference did not reach statistical significance (median 61.0 vs. 58.5 years). Sex distribution was comparable between groups. Notably, the AKI group exhibited a significantly higher prevalence of comorbidities, including hypertension, diabetes, COPD, cirrhosis, ARDS, heart failure, and respiratory failure. Furthermore, patients developing AKI required more frequent continuous renal replacement therapy and mechanical ventilation. Patients in the AKI group had a significantly lower baseline eGFR (median 52.0 vs. 82.0 mL/min/1.73m^2^, *p* < 0.001), indicating reduced renal functional reserve prior to the septic episode. With regard to laboratory indices, the AKI group displayed pronounced alterations in coagulation and inflammatory profiles. PTA was significantly lower (67.5% vs. 82.9%, *p* < 0.001), while TT, INR, FDP, D-D, and PT were elevated in AKI patients. Renal function markers, including blood urea nitrogen and Cr, were also higher in the AKI group. Inflammatory composite indicators differed markedly: the NLR, SII, and LAR were significantly higher, whereas the PNI, LMR, and AAPR were lower in the AKI group. The SOFA score was substantially higher in patients with AKI (median 10 vs. 7, *p* < 0.001).

**Table 1:** Baseline characteristics of the AKI group versus the non-AKI group.

Variable	Total (*N* = 362)	AKI (*N* = 206)	Non-AKI (*N* = 156)	*p*-value
Variables available at admission				
Age	60.00 [50.00, 72.00]	61.00 [51.00, 75.00]	58.50 [47.00, 69.50]	0.058
Gender				0.579
Male	225 (62.15%)	125 (60.68%)	100 (64.10%)	
Female	137 (37.85%)	81 (39.32%)	56 (35.90%)	
Temperature	36.50 [36.30, 37.00]	36.50 [36.30, 37.10]	36.50 [36.30, 36.90]	0.833
RR	20.00 [18.00, 22.00]	20.00 [18.00, 22.00]	20.00 [18.00, 22.00]	0.146
HR	89.0 [78.0, 106.0]	89.5 [80.0, 108.0]	89.0 [77.0, 105.0]	0.774
MAP	91.00 [78.00, 98.00]	91.17 [78.67, 97.67]	90.83 [77.67, 98.67]	0.486
Hypertension				<0.001
Yes	133 (36.74%)	103 (50.00%)	30 (19.23%)	
No	229 (63.26%)	103 (50.00%)	126 (80.77%)	
Diabetes				<0.001
No	256 (70.72%)	125 (60.68%)	131 (83.97%)	
Yes	106 (29.28%)	81 (39.32%)	25 (16.03%)	
CKD				0.012
Yes	52 (14.4%)	38 (18.4%)	14 (9.0%)	
No	310 (85.6%)	168 (81.6%)	142 (91.0%)	
COPD				<0.001
No	345 (95.30%)	189 (91.75%)	156 (100.00%)	
Yes	17 (4.70%)	17 (8.25%)	0 (0.00%)	
Cirrhosis				<0.001
No	290 (80.11%)	137 (66.50%)	153 (98.08%)	
Yes	72 (19.89%)	69 (33.50%)	3 (1.92%)	
ARDS				<0.001
No	308 (85.08%)	152 (73.79%)	156 (100.00%)	
Yes	54 (14.92%)	54 (26.21%)	0 (0.00%)	
Heart failure				<0.001
No	345 (95.30%)	189 (91.75%)	156 (100.00%)	
Yes	17 (4.70%)	17 (8.25%)	0 (0.00%)	
RF				<0.001
No	282 (77.90%)	136 (66.02%)	146 (93.59%)	
Yes	80 (22.10%)	70 (33.98%)	10 (6.41%)	
PTA	74.20 [54.00, 89.20]	67.50 [41.20, 85.00]	82.90 [67.80, 93.80]	<0.001
TT	17.10 [16.10, 19.00]	17.30 [16.30, 19.30]	16.70 [15.70, 18.25]	0.002
INR	1.28 [1.15, 1.41]	1.30 [1.20, 1.42]	1.25 [1.07, 1.40]	0.012
FDP	9.77 [4.70, 18.90]	11.20 [5.63, 20.82]	6.32 [3.29, 14.00]	<0.001
D-D	3.34 [1.80, 6.70]	3.60 [2.10, 6.80]	2.81 [1.40, 5.55]	0.021
FIB	3.95 [2.83, 5.29]	3.95 [2.47, 5.29]	3.98 [2.84, 5.36]	0.580
APTT	40.75 [36.50, 45.90]	41.60 [36.50, 45.90]	39.65 [36.50, 45.85]	0.292
PT	15.60 [14.30, 17.20]	16.10 [14.60, 17.30]	15.50 [13.90, 17.00]	0.040
AG Ratio	1.20 [1.00, 1.40]	1.20 [1.00, 1.50]	1.10 [1.00, 1.40]	0.099
Globulin	25.70 [21.60, 29.50]	24.30 [20.70, 28.50]	27.05 [23.15, 30.70]	<0.001
ALT	31.00 [20.00, 73.00]	32.50 [21.00, 78.00]	28.50 [19.00, 73.00]	0.351
AST	30.00 [16.00, 55.00]	28.00 [16.00, 51.00]	32.50 [16.00, 56.50]	0.890
Glucose	6.67 [5.21, 9.79]	7.12 [5.22, 10.43]	6.35 [4.90, 9.50]	0.262
Uric acid	285 [207, 382]	285.00 [213, 385]	282 [205, 371]	0.769
Cystatin C	1.18 [0.87, 2.25]	1.20 [0.87, 3.23]	1.17 [0.90, 1.75]	0.109
BUN	7.85 [4.91, 14.39]	9.24 [4.99, 19.66]	6.98 [4.91, 11.99]	0.013
Cr	70.5 [49.0, 154.0]	80.5 [49.0, 239.0]	70.0 [50.0, 100.5]	0.010
eGFR	68.5 [42.0, 95.0]	52.0 [30.0, 78.0]	82.0 [58.0, 105.0]	<0.001
TC	2.79 [1.86, 3.82]	2.74 [1.85, 3.67]	2.88 [1.96, 4.17]	0.143
PCT	1.62 [0.34, 12.00]	1.75 [0.40, 14.31]	1.33 [0.31, 6.97]	0.101
RDW-SD	49.10 [43.70, 56.00]	50.50 [45.30, 56.60]	47.05 [42.75, 54.30]	0.011
RDW-CV	14.40 [13.20, 16.00]	14.40 [13.40, 16.00]	14.40 [12.90, 16.10]	0.212
MPV	10.80 [10.00, 11.80]	10.80 [9.90, 11.80]	10.85 [10.00, 11.90]	0.429
CRP	68 [15, 115]	78 [31, 107]	47 [10, 119]	0.017
WBC	8.12 [5.99, 10.78]	8.20 [5.68, 10.41]	8.12 [6.29, 12.31]	0.404
Hemoglobin	106 [86, 131]	105 [76, 131]	109 [97, 130]	0.074
RBC	3.51 [2.79, 4.29]	3.44 [2.56, 4.31]	3.61 [3.09, 4.29]	0.035
AAPR	0.31 [0.17, 0.45]	0.27 [0.15, 0.41]	0.34 [0.23, 0.49]	<0.001
ALBI				0.065
3	132 (36.46%)	80 (38.83%)	52 (33.33%)	
2	190 (52.49%)	110 (53.40%)	80 (51.28%)	
1	40 (11.05%)	16 (7.77%)	24 (15.38%)	
NLR	10.50 [4.50, 22.03]	14.72 [6.51, 23.27]	5.63 [2.95, 16.31]	<0.001
PLR	175 [106, 317]	184 [102, 336]	164 [109, 269]	0.321
LMR	2.04 [1.03, 3.50]	1.84 [0.94, 3.11]	2.25 [1.17, 4.29]	0.027
SIRI	3.26 [1.05, 11.27]	3.26 [1.14, 12.41]	3.26 [0.84, 8.81]	0.135
PNI	34.41 [29.21, 40.46]	33.31 [27.76, 38.74]	35.83 [31.42, 42.24]	<0.001
SII	700 [184, 1,744]	1,170 [381, 2,143]	386 [98, 1,141]	<0.001
LAR	0.07 [0.04, 0.13]	0.09 [0.05, 0.17]	0.05 [0.03, 0.09]	<0.001
SOFA	9.00 [6.00, 10.00]	10.00 [7.00, 11.00]	7.00 [5.50, 9.00]	<0.001
Secondary outcomes and interventions during hospitalization				
Hospital LOS	14.00 [8.00, 23.00]	13.00 [7.00, 24.00]	15.00 [9.00, 23.00]	0.178
CRRT				<0.001
Yes	66 (18.23%)	66 (32.04%)	0 (0.00%)	
No	296 (81.77%)	140 (67.96%)	156 (100.00%)	
Mechanical ventilation				<0.001
Yes	63 (17.40%)	50 (24.27%)	13 (8.33%)	
No	299 (82.60%)	156 (75.73%)	143 (91.67%)	

RR, Respiratory rate; HR, heart rate; MAP, mean arterial pressure; ARDS, acute respiratory distress syndrome; RF, respiratory failure; COPD, chronic obstructive pulmonary disease; AKI, acute kidney injury; PTA, prothrombin activity; INR, international normalized ratio; PT, prothrombin time; APTT, activated partial thromboplastin time; ALP, alkaline phosphatase; AST, aspartate aminotransferase; ALT, alanine aminotransferase; D-D, D-dimer; FDP, fibrinogen degradation products; FIB, fibrinogen; UA, uric acid; BUN, blood urea nitrogen; Cr, creatinine; WBC, white blood cell count; PLT, platelet count; CRP, C-reactive protein; PCT, procalcitonin; RDW-CV, red cell distribution width-coefficient of variation; RDW-SD, red cell distribution width-standard deviation; MPV, mean platelet volume; NEUT, neutrophil count; RBC, red blood cell; lymphocyte count, TC, total cholesterol; SOFA, Sequential Organ Failure Assessment; CRRT, continuous renal replacement therapy; LOS, Length of stay; AAPR, Album-alkaline phosphatase ratio; ALBI, Albumin-bilirubin; NLR, Neutrophil-lymphocyte ratio; PLR, Platelet-to-lymphocyte ratio; LMR, Lymphocyte-to-monocyte ratio; SIRI, Systemic inflammation response index; PNI, Prognostic nutritional index; SII, Systemic immune-inflammation index; LAR, Lactate-albumin ratio.

### Feature selection and predictor identification

**Figure 2:**
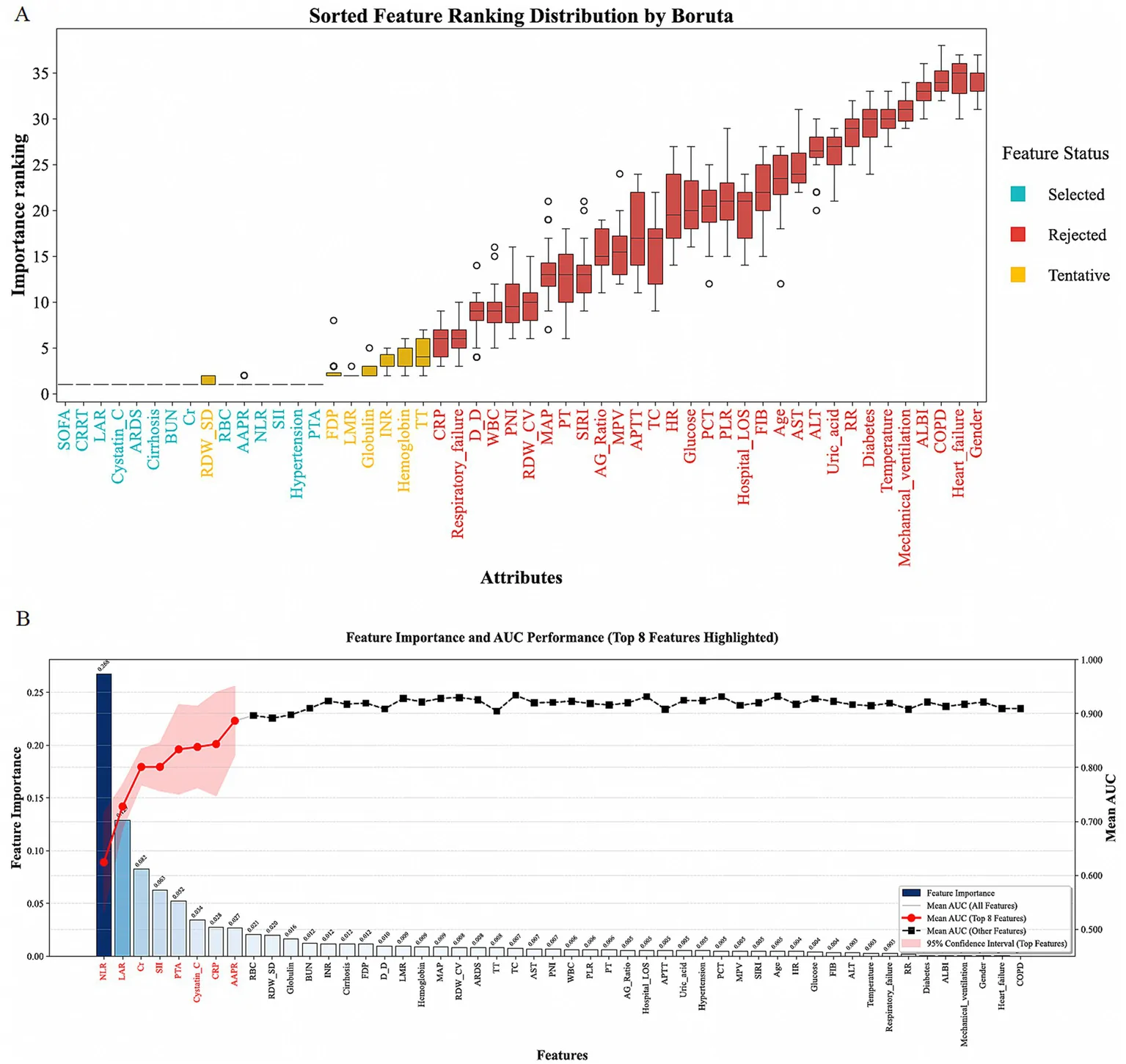
Identification of predictors for acute kidney injury in septic multiple myeloma patients. **(A)** Feature ranking by the Boruta algorithm, with selection outcomes denoted by color: green (confirmed important), yellow (tentative), and red (rejected). **(B)** Recursive feature elimination applied to the pre-selected candidate variables. The bar chart displays variables sorted by their contribution to the classification task, while the line graph traces the cumulative area under the receiver operating characteristic curve (AUC, right axis) as variables are added iteratively. The eight predictors highlighted in red were ultimately retained for model development.

To identify the most informative predictors while minimizing redundancy, we implemented a two-step feature selection pipeline. The Boruta algorithm initially identified multiple confirmed important variables (Figure 2A), predominantly comprising inflammatory and nutritional indices (NLR, SII, LAR, AAPR), renal function parameters (cystatin C, Cr, BUN), coagulation markers (PTA), comorbidities (hypertension, cirrhosis, ARDS), CRRT, RBC, and SOFA. Notably, because CRRT is a post-AKI therapeutic intervention rather than a baseline predictor, its inclusion would introduce information leakage and reverse causality. Therefore, CRRT was explicitly removed from the candidate variable pool prior to recursive feature elimination. Subsequently, recursive feature elimination determined that a compact set of eight variables achieved the highest AUC with the smallest number of predictors (Figure 2B). The final selected features were NLR, LAR, Cr, SII, PTA, cystatin C, CRP, and AAPR. Table 2 presents the distributions of these eight predictors across the training, test, and external validation cohorts. Significant inter-cohort differences were observed only for NLR (*p* = 0.022), while the remaining variables were comparable, indicating acceptable homogeneity and justifying the external validation strategy.

**Table 2:** Summary of selected predictors in the training set, test set, and temporal validation set.

Variable	Overall, *N* = 433	Training set, *N* = 253	Test set, *N* = 109	Temporal set, *N* = 71	*p*-value
NLR	9.46 [4.52, 21.28]	10.89 [4.66, 22.71]	8.89 [4.29, 19.01]	6.98 [4.82, 11.54]	0.022
LAR	0.07 [0.04, 0.13]	0.07 [0.04, 0.13]	0.07 [0.04, 0.14]	0.08 [0.05, 0.10]	0.787
Cr	71.0 [49.0, 152.0]	70.0 [49.0, 142.0]	74.0 [50.0, 169.0]	71.0 [49.0, 152.0]	0.897
SII	640 [206, 1,638]	616 [183, 1,608]	904 [206, 1,908]	566 [285, 1,516]	0.345
PTA	74.0 [54.0, 88.7]	74.0 [56.2, 90.0]	75.0 [50.3, 86.0]	73.0 [53.5, 86.0]	0.758
Cystatin C	1.22 [0.89, 2.38]	1.17 [0.87, 2.05]	1.27 [0.91, 2.63]	1.44 [0.98, 2.58]	0.096
CRP	66.7 [14.9, 118.5]	67.0 [12.6, 115.0]	63.1 [18.2, 103.5]	60.4 [10.3, 133.3]	0.994
AAPR	0.31 [0.17, 0.46]	0.31 [0.18, 0.46]	0.30 [0.17, 0.42]	0.34 [0.17, 0.49]	0.762

PTA, Prothrombin activity; Cr, creatinine; AAPR, Album-alkaline phosphatase ratio; NLR, Neutrophil-lymphocyte ratio; SII, Systemic immune-inflammation index; LAR, Lactate-albumin ratio; CRP, C-reactive protein.

### Performance comparison of machine learning models

The SVM and MLP models exhibited relatively modest discriminative ability, with temporal validation AUC values of 0.7244 and 0.5881, respectively. XGBoost achieved a high training AUC of 0.9458 but demonstrated a noticeable decline in the temporal validation cohort (AUC = 0.7764), suggesting moderate overfitting. LightGBM yielded perfect classification on the training set (AUC = 1.0) and retained acceptable temporal validation performance (AUC = 0.8229, F1 score = 0.7949), yet the near-perfect training metrics raised concerns regarding over-optimization.

**Figure 3:**
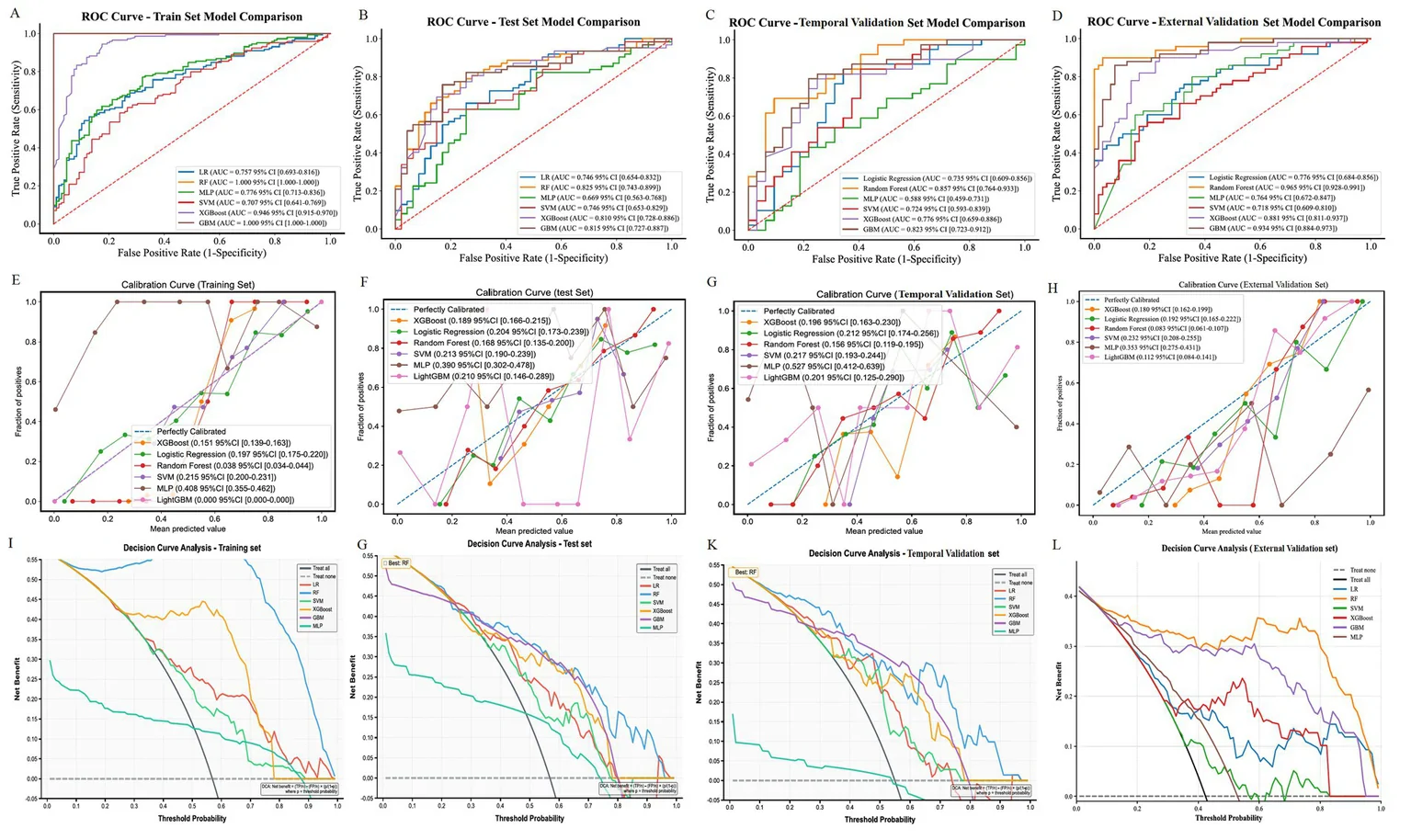
Comparative assessment of six predictive models. Discrimination, calibration, and clinical utility were evaluated by ROC curves **(A–D)**, calibration curves **(E–H)**, and decision curve analyses **(I–L)**, with the three graphs under each metric representing the training, test, temporal validation, and external validation sets, respectively.

Among all evaluated algorithms, Random Forest demonstrated the most robust and consistent predictive performance (Figures 3A–D). It achieved an AUC of 1.0 in the training cohort and maintained excellent discrimination in both internal test (AUC = 0.825, F1 score = 0.8031) and temporal validation (AUC = 0.8574, F1 score = 0.7805). Moreover, the model retained strong performance in the independent external validation cohort from Xi’an No. 3 Hospital (AUC = 0.9650, F1 score = 0.8824), further supporting its generalizability. Crucially, Random Forest exhibited the highest temporal validation AUC and the best balance between sensitivity (0.8205) and specificity (0.6562), translating into an accuracy of 0.7465 and precision of 0.7442. The minimal performance gap across training, internal test, temporal validation, and external validation underscored its superior generalizability compared with the other algorithms.

Calibration plots for all models and datasets are presented in Figures 3E–H. In addition to graphical calibration assessment, we quantified model calibration using the Brier score. In the temporal validation cohort, the Random Forest model achieved a Brier score of 0.156 (95% CI: 0.119–0.195), indicating good overall calibration without significant systematic over- or under-prediction. In the external validation cohort, the Brier score was 0.083 (95% CI: 0.061–0.107), further confirming the model’s reliability across different populations. Decision curve analysis (Figures 3I–L) confirmed that Random Forest provided a consistently positive net clinical benefit across a broad range of threshold probabilities, reinforcing its practical utility for risk-based clinical decision-making.

We further compared the predictive performance of our final Random Forest model with that of the SOFA score alone (Supplementary Figure 1S). In the temporal validation cohort, SOFA achieved an AUC of 0.652, which was significantly lower than that of our model (AUC = 0.857; *p* = 0.009, DeLong’s test). In the external validation cohort, the superiority of our model over SOFA was even more pronounced (AUC: 0.965 vs. 0.570; *p* < 0.001). These findings demonstrate that the eight composite inflammatory-nutritional indicators provide substantially better discrimination than the conventional SOFA score in this specific population (Table 3).

**Table 3:** Predictive performance of various machine learning algorithms evaluated on training, temporal validation, and external validation datasets.

Model	Dataset	Accuracy	Sensitivity	Precision	Specificity	F1 Score	AUC
SVM	Training	0.6561	0.7292	0.6863	0.5596	0.7071	0.7069
	Test	0.6606	0.7903	0.6712	0.4894	0.7259	0.7459
	Temporal validation	0.7042	0.7949	0.7045	0.5938	0.7470	0.7244
	External validation	0.6356	0.7600	0.5507	0.5441	0.6387	0.7176
XGBoost	Training	0.8577	0.9861	0.8068	0.6881	0.8875	0.9458
	Test	0.7339	0.8710	0.7200	0.5532	0.7883	0.8102
	Temporal validation	0.6901	0.8205	0.6809	0.5312	0.7442	0.7764
	External validation	0.7881	0.9000	0.6923	0.7059	0.7826	0.8809
LightGBM	Training	1.000	1.000	1.000	1.000	1.000	1.000
	Test	0.7615	0.8226	0.7727	0.6809	0.7969	0.815
	Temporal validation	0.7746	0.7949	0.7949	0.7500	0.7949	0.8229
	External validation	0.8644	0.8800	0.8148	0.8529	0.8462	0.9338
Logistic regression	Training	0.6957	0.7361	0.7310	0.6422	0.7336	0.7566
	Test	0.6606	0.7258	0.6923	0.5745	0.7087	0.7457
	Temporal validation	0.6901	0.6923	0.7297	0.6875	0.7105	0.7348
	External validation	0.7034	0.7000	0.6364	0.7059	0.6667	0.7765
Random forest	Training	0.9960	1.000	0.9931	0.9908	0.9965	1.000
	Test	0.7706	0.8226	0.7846	0.7021	0.8031	0.8250
	Temporal validation	0.7465	0.8205	0.7442	0.6562	0.7805	0.8574
	External validation	0.8983	0.9000	0.8654	0.8971	0.8824	0.9650
MLP	Training	0.5573	0.2569	0.8810	0.9541	0.3978	0.7756
	Test	0.5872	0.4032	0.7576	0.8298	0.5263	0.6692
	Temporal validation	0.4648	0.1026	0.5714	0.9062	0.1739	0.5881
	External validation	0.6186	0.9200	0.5287	0.3971	0.6715	0.7640

### Model interpretability via SHAP analysis

**Figure 4:**
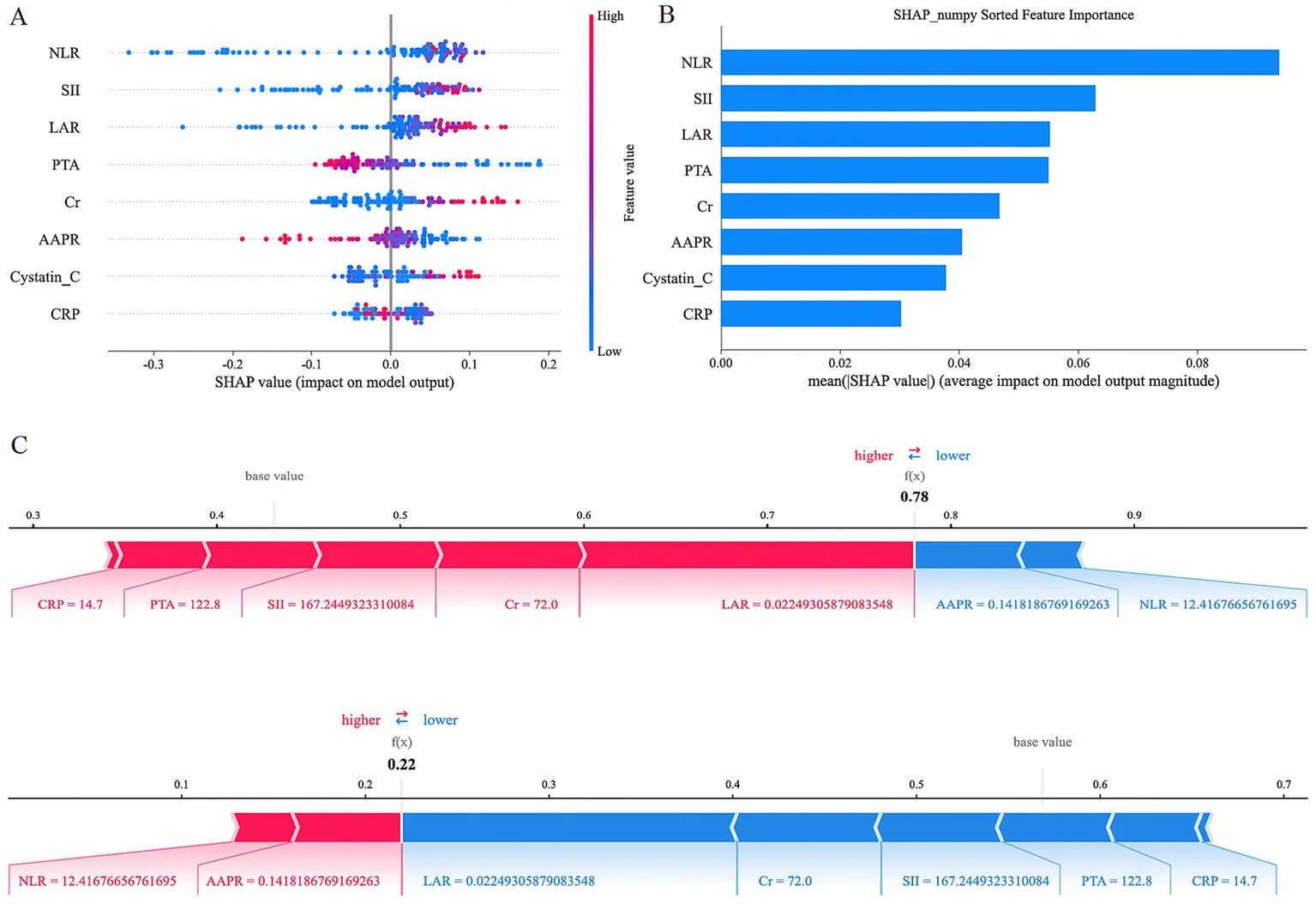
SHAP-based interpretation of the model **(A)** Summary plot of SHAP values, where the *x*-axis indicates the SHAP value, each row denotes a feature, and red/blue points represent high/low feature values, respectively. **(B)** Feature importance ranking produced by the logistic regression model. **(C)** Force plots illustrating the explanation of individual predictions for two independent cases.

To elucidate the driving factors behind individual risk predictions and ensure clinical transparency, we applied SHAP to the Random Forest model. Figure 4A displays the SHAP summary plot, which ranks features by their global importance and illustrates the direction of their influence on AKI prediction. The most influential features were NLR, LAR, SII, cystatin C, Cr, PTA, CRP, and AAPR. Higher values of NLR, LAR, SII, cystatin C, Cr, and CRP, as well as lower PTA and AAPR, were associated with higher SHAP values, indicating that these features contributed most strongly to the model’s predicted probability of AKI. The feature importance ranking derived from logistic regression (Figure 4B) demonstrated a broadly concordant pattern, corroborating the robustness of the selected predictors. Additionally, SHAP force plots (Figure 4C) provided patient-level explanations, illustrating how individual feature values shifted the model output from the baseline expectation. This dual-level interpretability—global feature ranking and local individual prediction decomposition—enables clinicians to understand and trust the model’s decision-making process.

### Deployment of an online prediction tool

**Figure 5:**
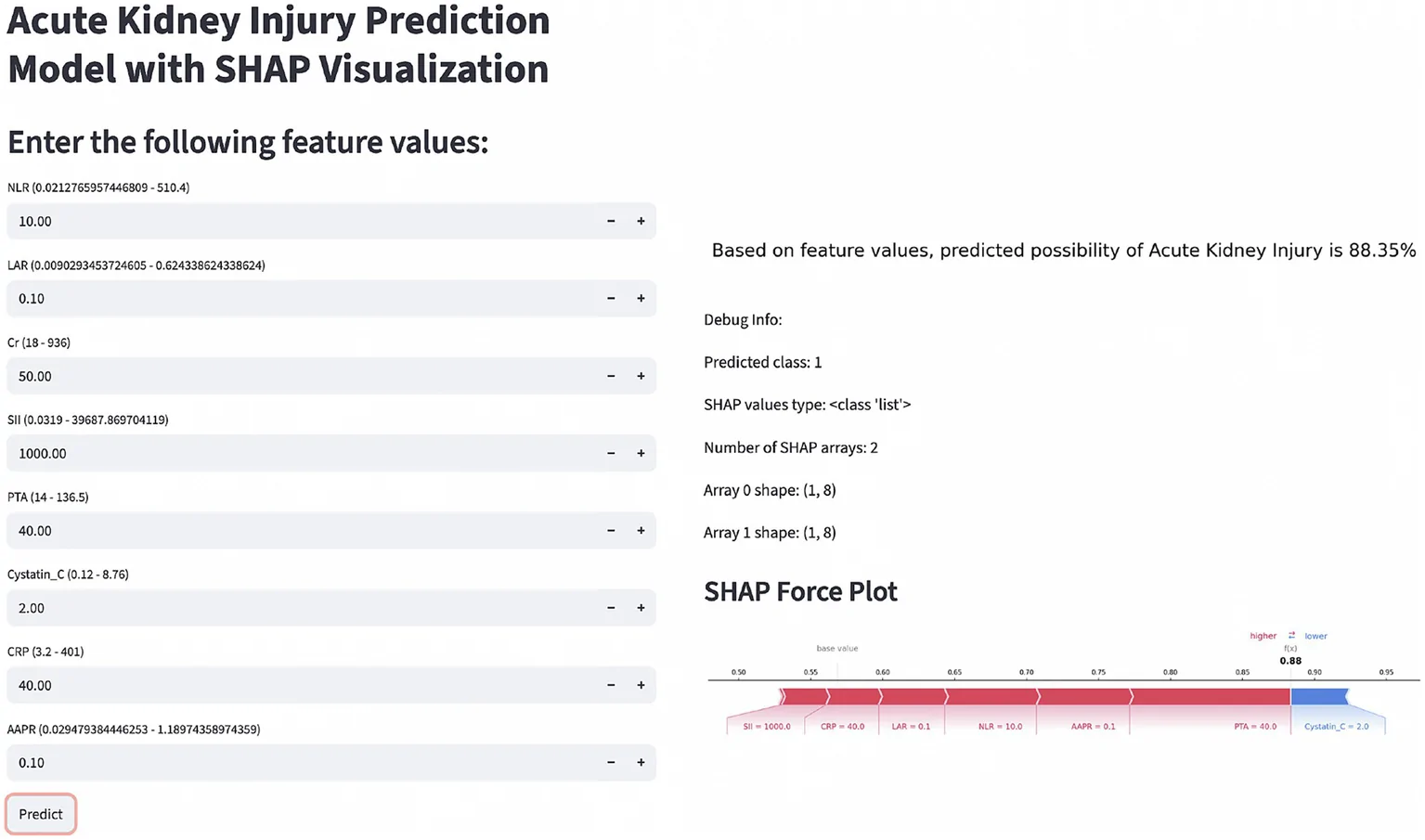
An online predictive tool for acute kidney injury in septic patients with multiple myeloma (https://gftcs94ast7omb44cg2izq.streamlit.app/).

To facilitate bedside application, the final Random Forest model was deployed as a freely accessible web-based prediction tool (Figure 5).^1^ This interface accepts the eight selected variables and instantly computes the individualized probability of AKI in septic patients with multiple myeloma, accompanied by visual explanations of feature contributions.

## Discussion

In this study, we developed an interpretable Random Forest model that predicts in-hospital AKI in septic patients with multiple myeloma using eight routinely available inflammatory and nutritional composite indicators. The model demonstrated robust performance in both temporal validation (AUC = 0.8574) and independent external validation (AUC = 0.9650), and was deployed as an open-access web application to support real-time, individualized risk assessment at the bedside. These findings suggest that a parsimonious set of laboratory-derived indices, when integrated into a carefully regularized machine learning framework, may effectively stratify renal risk in this vulnerable population, addressing a previously unmet need for tailored prediction tools in this dual-disease context.

The eight predictors retained by the Boruta-RFE pipeline—NLR, LAR, Cr, SII, PTA, cystatin C, CRP, and AAPR—collectively capture the intertwined pathophysiological axes that drive sepsis-associated AKI in the setting of MM. Systemic inflammation, reflected by elevated NLR, SII, and CRP, is a central mechanism of renal injury in sepsis. Prior work has demonstrated that the neutrophil-to-lymphocyte ratio, a marker of innate-to-adaptive immune imbalance, independently predicts the development of sepsis-induced AKI and its associated mortality (15). More recently, the systemic immune-inflammation index was shown to be a valuable, cost-effective predictor of both incident AKI and in-hospital death in septic patients (16). The reciprocal modulation of inflammation and metabolism in S-AKI, operating through NF-κB and TLR signaling, furnishes a mechanistic rationale for the pronounced prognostic signals captured by these integrative biomarkers (17). The particularly high AUC (0.965) observed in the independent external validation cohort warrants brief comment. As shown in Supplementary Table 1S, the AKI incidence in this cohort was 42.4%, considerably lower than in the development (56.9%) and temporal validation (54.9%) cohorts. This more balanced case–control distribution may have facilitated clearer separation between AKI and non-AKI patients, thereby yielding a higher absolute AUC. Additionally, the external cohort was derived from a different institution with potentially distinct patient referral patterns and disease severity profiles, which may have amplified the discriminative performance of the model. Nevertheless, this high AUC, combined with the consistent performance across temporal validation (AUC = 0.857), supports the model’s generalizability rather than suggesting overfitting.

Extending beyond pure inflammatory markers, the lactate-to-albumin ratio integrates tissue hypoperfusion with the host nutritional and inflammatory reserve. Karampela et al. demonstrated that LAR measured at ICU admission was significantly higher in septic shock patients and non-survivors, and that an elevated baseline LAR independently predicted 28-day mortality (HR 2.27, *p* = 0.04) (18). In our cohort, the elevated LAR observed among patients who subsequently developed AKI suggests that the composite metric captures not only hemodynamic instability but also the degree of pre-existing physiological fragility—an insight that single markers such as lactate or albumin alone may fail to convey. Complementing this profile, the albumin-alkaline phosphatase ratio was selected as a composite indicator of nutritional status and hepatobiliary integrity. Liu et al. recently demonstrated that an elevated alkaline phosphatase-to-albumin ratio is significantly associated with 90-day and 1-year mortality in sepsis, with AUCs approaching 0.71 after adjustment for confounders (19); correspondingly, lower AAPR was previously linked to increased in-hospital and 30-day mortality among critically ill patients with AKI (20). The AAPR’s incremental value in our model aligns with the growing recognition that nutritional-inflammatory crosstalk is a modifiable determinant of renal outcomes.

The inclusion of PTA and the renal functional markers Cr and cystatin C further reflect the pathognomonic convergence of immunothrombosis and subclinical renal vulnerability in this dual-disease population. Sepsis drives a consumptive coagulopathy characterized by reduced prothrombin activity and simultaneous microvascular fibrin deposition; PTA decline thus signals both hepatic synthetic impairment and endothelial injury within the renal microcirculation. While creatinine is also used in the KDIGO criteria for AKI diagnosis, its role as a baseline predictor in our model should be interpreted as a marker of pre-existing renal functional reserve rather than as a diagnostic criterion for the outcome event. By excluding patients with AKI at admission or within the first 24 h, we ensured that the creatinine value used for prediction reflected the patient’s baseline kidney function prior to the septic insult, rather than an established AKI episode. Similarly, cystatin C—a marker less influenced by muscle mass and more sensitive to early glomerular filtration changes—was included to capture subclinical renal vulnerability that may predispose patients to subsequent AKI when challenged by sepsis. Meanwhile, the baseline elevation of Cr and cystatin C prior to overt KDIGO-defined AKI suggests that a proportion of septic MM patients already harbor incipient tubular injury or diminished renal functional reserve at presentation. This observation resonates with the concept that monoclonal free light chains constitutively induce proximal tubular inflammation through NF-κB-dependent transcription of cytokines such as IL-6 and MCP-1, and bind to Tamm-Horsfall protein in the distal nephron to form obstructing casts, thereby priming the kidney for additional septic insults (21). It should be noted that while we refer to the admission creatinine as the ‘baseline’ for AKI definition, this value may not reflect the patient’s true pre-morbid renal function, as chronic kidney disease or myeloma-related tubular injury may already be present at admission.

A legitimate concern is that our model does not directly incorporate MM-specific variables, such as serum free light chains, Durie-Salmon stage, or prior antimyeloma therapy. We acknowledge that these factors undeniably modulate renal vulnerability. Nevertheless, we argue that the selected eight predictors effectively capture the functional consequences and final common pathways of both myeloma- and sepsis-induced renal insults. For instance, while sFLC levels are the primary driver of cast nephropathy, their nephrotoxic effect is ultimately reflected by the baseline creatinine and cystatin C levels, which represent the kidney’s functional reserve and subclinical tubular injury prior to the septic event. Moreover, the degree of monoclonal protein burden may be indirectly mirrored by the serum globulin fraction and the albumin-to-alkaline phosphatase ratio (AAPR), which integrates both nutritional reserve and hepatobiliary/plasma cell turnover. Regarding septic shock and vasopressor exposure, although not directly included, the lactate-to-albumin ratio (LAR) and SOFA score components encapsulate tissue hypoperfusion and multiorgan dysfunction, which are the immediate mechanistic links between hemodynamic instability and AKI. Thus, rather than dissecting the precise etiology of AKI (myeloma cast nephropathy vs. septic acute tubular necrosis), our model serves as a pragmatic, unified risk stratification tool that identifies patients who are already at the convergence point of these two pathological cascades, enabling timely renoprotective actions before definitive etiological workups are completed.

From a modeling perspective, the relative performance of Random Forest compared with LightGBM and XGBoost in our evaluation merits careful interpretation. Although LightGBM yielded a perfect training AUC, its temporal validation performance (AUC = 0.8229) indicated over-optimization; XGBoost similarly showed a notable gap between training (AUC = 0.9458) and temporal validation (AUC = 0.7764) performance. In contrast, Random Forest demonstrated more stable performance across internal test (AUC = 0.825), temporal validation (AUC = 0.8574), and independent external validation (AUC = 0.9650) cohorts, and achieved the highest temporal validation F1 score (0.7805) with a clinically favorable balance of sensitivity and specificity. These findings echo recent work by Zhen et al., who developed explainable machine learning models for AKI staging in sepsis patients and likewise found Random Forest to exhibit optimal and stable performance compared with gradient boosting and neural network alternatives (22). In a scoping review of 25 ML-based S-AKI prediction models, Li et al. noted that most published models lacked external validation and that random forest and gradient boosting methods offered comparable discriminative performance, yet the former tended to exhibit more resilience against overfitting in moderate-sized datasets (4). More broadly, the ORAKLE deep-learning study demonstrated that incorporating dynamic time-series data can enhance AKI outcome prediction (23); our model, while constrained to static admission data, benefits from the biological richness of composite inflammatory-nutritional indices and the parsimony of an eight-variable input, which together confer both predictive accuracy and practical feasibility at the point of care.

A defining feature of our framework is the integration of SHAP-based interpretability, which transforms the Random Forest model from a black-box predictor into a clinically transparent decision-support instrument. The summary plots and individual force plots reveal not only which features are most influential in the model globally, but also how each feature value shifts a given patient’s predicted probability away from the baseline expectation. It is important to note that SHAP values describe contributions to model predictions and should not be interpreted as evidence of causal effects or biological mechanisms, but rather as a tool for understanding the model’s decision-making process. The concordance between SHAP-derived importance and logistic regression coefficients further validates the model’s internal consistency. This dual-level transparency has been identified as a critical enabler of clinician trust in AI-augmented decision-making. Vani et al. recently argued that explainable AI frameworks that offer patient-specific, model-consistent explanations are essential for bridging the gap between algorithmic complexity and clinical acceptance (24), and Hur et al. provided empirical evidence that clinicians’ acceptance and trust of AI-powered clinical decision support systems are significantly modulated by the quality and format of the explanations provided (25).

The clinical significance of our model lies in its capacity to provide early, individualized AKI risk estimates using predictors that are routinely available within the first 24 h of ICU admission. The decision curve analysis confirmed that Random Forest delivered a consistent net clinical benefit across a broad range of threshold probabilities, which is particularly important in a population where the observed AKI incidence exceeded 50%. In such a high-prevalence setting, a “treat-all” approach to renal protection would expose the majority of patients to unnecessary interventions and potential iatrogenic harm; a risk-stratified strategy guided by our model could more precisely allocate intensive monitoring, nephrology consultation, and renoprotective measures to patients most likely to benefit. Although CRRT was ultimately required in nearly one-third of patients who developed AKI, this therapeutic intervention was intentionally excluded from the predictor pool to avoid reverse causality. By identifying high-risk individuals before overt renal injury manifests, the model offers a window for proactive intervention that may ultimately reduce the need for dialytic support.

Building on these pathophysiological considerations and the evidence base reviewed above, the composite indicators identified in this study—spanning systemic inflammation, metabolic stress, nutritional reserve, and coagulation function—collectively reflect the multilevel mechanisms through which sepsis precipitates renal injury in patients with MM. The external validation results, the favorable decision curve analysis, and the SHAP-based interpretability together support the potential clinical utility of this tool for early risk stratification. The integrated workflow from feature selection to web-based deployment further illustrates the feasibility and value of translating complex machine learning models into accessible point-of-care applications for this high-risk population.

## Limitations

Several limitations of this study merit acknowledgment. First, the temporal validation cohort was derived from the same institution, which may introduce institutional bias despite temporal separation. However, the additional independent external validation from Xi’an No. 3 Hospital partially mitigates this concern. Second, AKI was defined by serum Cr changes per KDIGO criteria rather than by systematic screening protocols, which may have led to under-detection of non-oliguric episodes. Additionally, the absence of systematically documented pre-admission serum creatinine values means that the ‘baseline’ creatinine used in our analysis was derived from the first measurement after admission rather than a true pre-morbid value. This may have led to misclassification of AKI in patients with pre-existing renal impairment, although this limitation is inherent to retrospective studies using KDIGO criteria and is unlikely to have differentially affected our comparison groups. Third, while our model predicts the overall risk of AKI in septic patients with MM, the specific etiology of kidney injury—whether primarily sepsis-associated, myeloma-related cast nephropathy, or drug-induced—could not be definitively differentiated using this retrospective dataset. We acknowledge that in this population, AKI is often multifactorial, and our intention is to provide a practical risk-stratification tool to guide early monitoring and renoprotective measures, rather than to establish causal pathways. The absence of detailed data on antimyeloma therapy (e.g., proteasome inhibitors, immunomodulatory drugs), serum free light chain levels, and urinary biomarkers of tubular injury represents an important direction for future research to refine the model and better disentangle these overlapping mechanisms. Fourth, the sample size, although adequate for model development and internal validation, limited the statistical power for subgroup analyses. Finally, while the model was deployed as an online tool, prospective implementation studies are required to evaluate its real-world impact on clinical workflows, clinician behavior, and patient-centered renal outcomes.

## Conclusion

In summary, we developed, externally validated, and deployed an interpretable Random Forest-based model for predicting in-hospital AKI in septic patients with MM using eight inflammatory and nutritional composite indicators. The model demonstrated robust discrimination, satisfactory calibration, and positive net clinical benefit, and was encapsulated into a freely accessible web application. By leveraging SHAP-based explainability, our framework bridges the gap between algorithmic complexity and clinical transparency, establishing the groundwork for individualized renal protective strategies in this high-risk population.

## Data Availability

The raw data supporting the conclusions of this article will be made available by the authors, without undue reservation.

## Footnotes

1 https://gftcs94ast7omb44cg2izq.streamlit.app/.
