# Supplementary material for: A machine-learning-derived online prediction model based on inflammatory and nutritional composite indicators for acute kidney injury in sepsis patients with multiple myeloma
Source: Front Nutr. 2026 Sep 7;13:1874801. doi: 10.3389/fnut.2026.1874801 (PMC13590401; doi:10.3389/fnut.2026.1874801)
Supplement: Supplementary file 1 [file Supplementary_file_1.DOCX]

**Table 1S.** Incidence, KDIGO stage, and timing of AKI across the training, internal test, and temporal validation cohorts

| Cohort | N | AKI incidence | KDIGO stage 1 | KDIGO stage 2 | KDIGO stage 3 | Time to AKI onset (days) |
| --- | --- | --- | --- | --- | --- | --- |
| Training | 253 | 144 (56.9%) | 76 (52.8%) | 38 (26.4%) | 30 (20.8%) | 5.0 (3.0–8.0) |
| Internal test | 109 | 62 (56.9%) | 33 (53.2%) | 16 (25.8%) | 13 (21.0%) | 5.0 (3.0–8.0) |
| Temporal validation | 71 | 39 (54.9%) | 21 (53.8%) | 10 (25.6%) | 8 (20.5%) | 5.0 (3.0–8.0) |
| External validation | 118 | 50 (42.4%) | 27 (54.0%) | 13 (26.0%) | 10 (20.0%) | 6.0 (3.0–9.0) |

Data are presented as n (%) unless otherwise specified. KDIGO stages were defined according to the Kidney Disease: Improving Global Outcomes criteria. Time to AKI onset was calculated from hospital admission to the first documentation of KDIGO criteria fulfillment. The temporal validation cohort comprised patients admitted to the same institution between November 2024 and December 2025. The external validation cohort comprised patients admitted to a separate tertiary hospital between November 2023 and December 2025.

**Figure 1S.** Assessment of SOFA score for AKI prediction. (A) SOFA score in the temporal validation set (AUC = 0.652). (B) SOFA score in the external validation set (AUC = 0.570).
